# Advances in Cell Transplantation Therapy for Diseased Myocardium

**DOI:** 10.4061/2011/679171

**Published:** 2011-06-28

**Authors:** Outi M. Villet, Antti Siltanen, Tommi Pätilä, M. Ali A. Mahar, Antti Vento, Esko Kankuri, Ari Harjula

**Affiliations:** ^1^Department of Cardiothoracic Surgery, University of Helsinki Meilahti Hospital, P.O. Box 340, FIN-00029 HUS, Finland; ^2^Department of Pharmacology, Institute of Biomedicine, University of Helsinki, 00014 Helsinki, Finland

## Abstract

The overall objective of cell transplantation is to repopulate postinfarction scar with contractile cells, thus improving systolic function, and to prevent or to regress the remodeling process. Direct implantation of isolated myoblasts, cardiomyocytes, and bone-marrow-derived cells has shown prospect for improved cardiac performance in several animal models and patients suffering from heart failure. However, direct implantation of cultured cells can lead to major cell loss by leakage and cell death, inappropriate integration and proliferation, and cardiac arrhythmia. To resolve these problems an approach using 3-dimensional tissue-engineered cell constructs has been investigated. Cell engineering technology has enabled scaffold-free sheet development including generation of communication between cell graft and host tissue, creation of organized microvascular network, and relatively long-term survival after *in vivo* transplantation.

## 1. Introduction

Cardiac repair by cell therapy offers hope to improve performance of diseased heart by reconstituting or maintaining cardiac specific tissue [1]. First studies were performed with cells such as myoblasts [2], but later the field expanded to several cell types including bone marrow cells [3], endothelial progenitors [4], mesenchymal stem cells (MSCs) [5], resident cardiac stem cells [6], and embryonic stem cells [7]. Numerous preclinical studies have shown improved cardiac function in animal models of heart failure, but the underlying mechanisms of this improvement have remained obscure.

Nevertheless, the hypothesis that cardiac function in heart failure can benefit from cell therapy has gained extensive attention and preliminary clinical trials have been launched [8–14]. Miyagawa et al., reported their first clinical trial with combined cell therapy instead of single cell therapy for a patient based on their finding that combined cell therapy (both myoblasts and bone marrow mononuclear cells) has a more synergistic effect on severely damaged myocardium [15]. In this case recovery of cardiac function and histological changes were observed. However, they could not establish if the dramatic functional improvement was attributable to decreased left ventricle (LV) distension by a left ventricular assist system (LVAS) or to the cell transplantation due to lack of an appropriate control. The brain natriuretic peptide (BNP) levels were significantly lower after cell transplantation than under LVAS before cell transplantation [15]. However, for those patients with ischemic cardiomyopathy, LVAS implantation alone does not achieve sufficient recovery of myocardial function. After LVAS implantation, decreased LV distension contributed to the reduced cell diameter and lower BNP [15]. They also detected other changes such as improved regional diastolic function and vascular density in the targeted region, in addition to the changes evoked by LVAS implantation. This indicates that cell transplantation had a positive effect on the distressed ischemic myocardium. Yet, the mixed results received from the clinical studies have regained the interests for laboratory work.

## 2. Cell Delivery by Injection

One of the crucial issues in cell therapy for heart failure has been the cell delivery route. Injection of the cells has been the most typical method in clinical feasibility studies (Table 1). The cells have been injected directly into the myocardium or into the coronary vasculature. However, several underlying issues make this a challenging technique to use clinically. The injected cells can be washed out through channel leakage and the vascular system, creating a significant loss of grafted cells [16–18]. Additional loss of the grafted cells is generated by the disruption of the extracellular matrix and the subsequent loss of signals that modulate cell survival, differentiation, and patterning [19].

In addition, arrhythmogenicity of intramyocardial myoblast transplantation has aroused debate. Episodes of ventricular tachycardia and fibrillation have been noted after several feasibility studies. Due to small number of patients in nonrandomized series and the arrhythmogenic nature of the heart disease itself, direct causality is difficult to conclude. In an early study of Menasché et al., 4 out of 10 patients experienced sustained ventricular tachycardia [22], and in the study by Smits et al., 4 out of 15 patients experienced clinically significant arrhythmias in the early phase [20]. A four-year surveillance of the patients showed more intracardiac defibrillator (ICD) interventions when compared to a matched patient population [21]. According to the preliminary data, all patients in MAGIC trial received an ICD implant. At one year after the myoblast injections, no significant difference was found between the myoblast-treated group or the control group regarding arrhythmogenicity [14]. To overcome this evident problem, several strategies for preventing cell therapy-associated arrhythmias have been introduced. Genetic engineering by overexpressing connexin-43 by the transplanted cells might improve the cell graft integration to the host [23]. Myocardial damage resulting from multiple needle injections is potentially a significant cause to ventricular arrhythmias. Avoiding the injections by using intracoronary delivery might decrease arrhythmia [24], although cell loss has been described [25]. Still, even more important aspect would be to improve the method of cell delivery. Various cell types and their delivery methods into damaged myocardium are compared in Table 2.

## 3. Cell Delivery by Injection versus Cell Sheets

Another approach for cell delivery as opposed to injection involves tissue-engineered constructs. The main advantage of this technology over standard cell implantation lies on the preservation of microcellular communication and matrix, which is lost upon trypsin treatment in the typical procedure of cell preparation. Epicardial deposition of cell sheets might be a solution to prevent significant cell loss and arrhythmia after cell transplantation [26]. Memon et al., reported that myoblast sheet implantation improved global cardiac function to a greater extent than the injection of cell suspension [27]. Similarly, in a recent study, myoblast cell sheets were compared to direct injections of myoblast cells in ischemic heart failure (Patila et al., submitted). Holter monitoring showed more ventricular premature contractions in the cell injection group. Furthermore, epicardial electropotential mapping showed areas of electrical reentry in the injection sites. RT-PCR showed more inflammatory markers and more inflammatory cells accumulated at the infarct border area in the injection group, when compared to the cell sheet-treated animals (Patila et al., submitted).

## 4. Cell Sheets in Diseased Heart

Current tissue engineering methods allow us to reconstruct myocardial tissue grafts for clinical applications, though human fetal and neonatal cardiomyocytes are difficult to obtain. Therefore, several classes of stem cells are being investigated as a potential cell source. Despite their attractive potential to differentiate into various cell types, several issues remain, including difficulties in obtaining and amplifying the cells and the lack of understanding of the mechanisms for differentiation and proliferation. Consequently, the clinical cell sheet transplantation has mainly focused on utilization of myoblasts.

Memon et al., [27] reported the use of skeletal myoblast sheets in a rat myocardial infarction model. Preclinical data have shown that autologous skeletal myoblasts are capable not only of fusion and differentiation into striated muscle cells within damaged myocardium [31] but also of augmenting systolic and diastolic performance in animal models of acute myocardial infarction and heart failure [28–30, 46]. Inhibition of apoptosis in myoblast sheets by expression of antiapoptotic *bcl2 *was shown to enhance the efficacy of sheet transplantation therapy in acute myocardial infarction [43]. Moreover, Siltanen et al. demonstrated that prevention of graft apoptosis by *bcl2 *improved myoblast sheet transplantation therapy also in chronic myocardial infarction model [44].

Dilated cardiomyopathy (DCM) is characterized by global myocardial remodeling, which mainly consists of myocardial fibrosis associated with changes in the cytoskeletal and sarcolemmal proteins in cardiomyocytes, leading to a reduction in the number and function of these cells [47]. Consequently, cardiac remodeling chronically progresses with ventricular dilation and thinning, leading to progressive congestive heart failure. Kondoh et al. used human hereditary DCM representative model of TO-2 hamster strain [32]. In this hamster strain, the number of cardiomyocytes decreases progressively because of apoptosis after birth, and cardiac remodeling, which mainly consists of myocardial fibrosis, occurs with ventricular dilation and thinning, leading to progressive congestive heart failure. Transplantation of myoblast sheet grafts showed reorganization of the cytoskeletal proteins, reduction of myocardial fibrosis, and prevention of dilation of the left ventricle, leading to prolonged life expectancy and a longer preservation of cardiac performance in the impaired heart.

Hoashi et al., [42] showed that myoblast sheet transplantation improved right ventricular diastolic dysfunction. Briefly, animals underwent pulmonary artery banding thus created chronic pressure overload resulting in right ventricular failure. Chronic pressure overload is one of the major risk factors of right ventricular dysfunction. In this situation the right ventricle is hypertrophied and systolic function is initially preserved, whereas diastolic function gradually deteriorates. Prolonged exposure to excessive pressure overload results in irreversible right ventricular failure. Thus they successfully demonstrated improvement in diastolic dysfunction and suppressed ventricular fibrosis with increased capillary density in a rat model of a pressure-overloaded right ventricle by implanting myoblast sheets.

Preclinical studies in impaired porcine heart using single monolayer skeletal muscle cell sheet demonstrated improved cardiac performance accompanied with increased myocardial perfusion and viable myocardial tissue [45].

Further, Miyahara et al., used mesenchymal stem cells (MSCs) derived from adipose tissue in a rat myocardial infarction model [33]. MSC sheets incorporated into the host myocardium and improved cardiac function and increased survival. Similarly, Okura et al., showed that transplanted sheets of adipose tissue-derived MSCs differentiated into cardiomyoblast-like cells and resulted in recovery of cardiac function and improved survival rate of rat with infarcted heart [34].

## 5. Development of Complex Sheet Structures

Difficulties still exist in the outcome of cell therapy, as it is challenging to control the cell growth and localization of the grafted cells and to deliver a cell sheet patch that significantly aids the function of the damaged myocardium. To overcome these problems research has begun on fabricating three-dimensional cardiac grafts composed of multilayered cell sheets. Several methods have been studied with reconstructed tissues based on biodegradable scaffolds, such as poly(lactic-co-glycolic acid) and gelatin or extracellular matrix components [48, 49].

### 5.1. Fabrication of Scaffold-Free Cell Sheets

In native cardiac tissue the cell density is considerably high, cells being tightly interconnected with gap junctions facilitating electrical communication. The use of scaffolds can lead to abnormal tissue development, electrical communication caused by insufficient cell-to-cell connections, inflammatory responses, and fibrous tissue formation. Alternatively, fabrication of scaffold-free cell sheets requires means of cell detachment from the culture surface that will preserve cell morphology, orientation within the scaffold, and adhesion to surrounding cells and the extracellular matrix. One way to achieve such detachment is to covalently employ a temperature-responsive polymer on cell culture surface. Poly(N-isopropylacrylamide) (PIPAAm) is a hydrophobic polymer at temperatures above 32°C which—after grafting to cell culture dishes—allows cell adhesion and proliferation [50]. At temperatures below 32°C, PIPAAm grafted surfaces change their properties and become hydrophilic to allow cell detachment as intact sheets which harbor the ECM on the basal surface [51]. These sheets retain their cell-to-cell as well as cell-to-ECM adhesions [51], while cell viability is not compromised [50]. Further, PIPAAm-grafted surfaces can be used to engineer monolayer [52] as well as three-dimensional sheet structures comprised of several cell layers [53].

Stevens et al., and Itabashi et al., described other methods for creating scaffold-free sheets. Stevens et al., created embryonic stem cell-derived cardiomyocyte sheets utilizing Teflon-coated low-attachment tissue culture dishes combined with rotating orbital shaker [54]. The diameter of these sheets was dependent on cell number, and the thickness was approximately 300–600 *μ*m. These sheets, however, were subject to necrosis due to limited oxygen supply. The same method was then used to create prevascularized sheets composed of cardiomyocytes, endothelial cells, and fibroblasts. These sheets effectively integrated with the coronary circulation after implantation and evaded necrosis. Furthermore, Itabashi et al., fabricated cardiomyocyte sheets using polymerized fibrin-coated culture dishes [55]. This method is based on the proteolytic activity of the cardiomyocytes that degrades the underlying fibrin coating and allows harvesting of intact sheets mechanically using a cell scraper.

### 5.2. Cellular Communication in Cell Sheets

In addition to cell-to-cell communication, the layers need to establish a connection with each other and with the host tissue. Using a multielectrode extracellular recording system, Haraguchi et al., demonstrated that the electrical coupling between 2 sheets starts approximately 34 minutes after initial layering and is completed by about 46 minutes. They also showed small molecule exchange through gap junctions and presence of connexin-43 within 30 minutes of layering [56].

When Shimizu et al., implanted a 4-layered neonatal rat cardiomyocyte sheet into the subcutaneous space of nude rats, synchronous beating [57] and survival up to 1 year [58] were observed. The implanted graft showed characteristic structures of heart tissue, including elongated cardiomyocytes, well-differentiated sarcomeres, and gap junctions. Additionally, conduction velocity, contractile force, and size of implanted grafts increased in proportion to the host's growth [58]. Hata et al., further showed synchronous contraction with defined direction of neonatal rat cardiomyocyte sheets on decellularised porcine small-intestinal submucosa [59].

Miyagawa and colleagues showed that neonatal cardiomyocyte sheets fabricated on temperature-responsive culture dishes attached to the infarcted myocardium and led to an improvement in cardiac performance and improved vascular density [37]. The implanted sheets communicated with the host myocardium as indicated by the presence of connexin-43 and changes in the QRS wave and action potential amplitude.

Another study demonstrated a similar electrical integration between a neonatal myocyte sheet and the host myocardium without serious arrhythmia [26]. Furthermore, histological analyses in infarcted rat hearts with a transplanted 3D tissue graft showed bridging of the cardiomyocytes with functional gap junctions and intercalated disks [38].

### 5.3. Recreation of Microvessels and Cell Sheet Survival

Heart is a metabolically active organ that requires virtually constant oxygen supply in order to function in a normal fashion. The major limitation of the multilayered cell grafts is insufficient circulation causing hypoxia, nutrient insufficiency, and accumulation of waste products. Cells in living tissues receive oxygen supply through a capillary network, whereas the cultured cell aggregates *in vitro* rely on diffusion. In order to reconstruct thicker and metabolically active tissue grafts sufficient blood supply network has to be created.

One strategy could be to generate capillary-like networks *in vitro *in tissue-engineered constructs before transplantation. Levenberg et al., demonstrated the induction of endothelial vessel networks in engineered skeletal muscle tissue constructs using a 3D culture system consisting of myoblasts, embryonic fibroblasts, and endothelial cells coseeded on porous, biodegradable polymer scaffolds [60]. Similar spontaneous 3D prevascular network formation has been shown also in *in vitro* bone coculture model with human MSCs, human umbilical vein endothelial cells (HUVECs) [61, 62], and in human endothelialized reconstructed skin (ERS), including keratinocytes, fibroblasts, and endothelial cells in a collagen sponge [63]. As the reconstructed skin was transplanted to a nude mouse, Tremblay et al., concluded that the early vascularization observed in the ERS was most likely the result of inosculation of the capillary-like structures with the host's capillaries, rather than neovascularisation, which is a slower process.

Sasagawa et al., developed a novel cell sheet stacking manipulation technique to create multilayered cell sheets from human skeletal muscle myoblasts [64]. They placed a hydrogel-coated plunger onto a confluently cultured cell layer in a temperature-responsive culture dish. To harvest the cell layer the temperature was decreased to 20°C after which the plunger with the cell layer was transferred onto another confluent myoblast monolayer in another dish and incubated at 37°C to promote the cell layer adhesion. After 30–50 minutes in 20°C the plunger was lifted up with a double-layer myoblast sheet. They were able to reach a 5-layered construct with this procedure, which did no harm to the cells confirmed by cell viability assay. To further develop a viable cell dense tissue construct Sasagawa et al., sandwiched HUVECs into the 5-layer myoblast sheet construct. Four days after culture, the HUVECs had started to develop into capillary-like structures. One week after the constructs were engrafted on the dorsal subcutaneous tissue of nude rat, newly formed microvessels connected to the host vessels were found.

A single myoblast layer is about 45 *μ*m thick [57]. Accordingly, most previous studies used multilayer constructs up to about 250 *μ*m thick. In order to progress the human cell sheet applications the greater thickness of the construct would be of great advantage. Sekiya et al., investigated the relationship between the number of transplanted cell layers and cardiac function. They found a significant improvement of cardiac function, induction of angiogenesis, more elastic fibers, and less fibrosis with implantation of 3- and 5- layered myoblast sheets compared to single layer [41].

Shimizu et al., showed that the 1-, 2-, and 3-layer constructs transplanted into the dorsal subcutaneous tissue of nude rats thoroughly survived without necrosis [39]. However, parts of the 4- and 5-layer constructs showed disordered vasculature and connective tissue, indicative of necrosis. Subsequently, transplantation of two triple-layer grafts at 1-day intervals permitted whole tissue survival with a well-organized microvascular network, whereas 2- and 3-day transplantation intervals had poor outcome. Shimizu et al., further developed the polysurgical method by transplanting up to 10 triple-layer cell sheet grafts at 1- or 2-day intervals. Interestingly, at one week after the final transplantation about 0.9 mm thick cell-dense myocardium graft revealed vigorous myocardium-like pulsation with well-organized microvessels throughout the graft. This polysurgical method would encounter difficulties in clinical replacement therapy as each procedure has a relatively high risk of complications. To overcome this obstacle, Shimizu et al., developed an ectopic construct repeatedly transplanting the layered cell sheets over an exposed superficial caudal epigastric, and femoral artery and vein of a nude rat. After 2 weeks the pulsating graft was resected together with the femoral artery and vein and further connected to carotid artery and the jugular vein in a new host. The grafts survived and maintained their characteristic beating 2 weeks after the procedure.

### 5.4. Angiogenesis in Cell Sheets

One way to enhance the cell sheet graft survival is to promote angiogenesis. Enhanced angiogenesis and functional improvement were achieved by using cocultured cell sheets with fibroblasts and endothelial progenitor cells [40]. Another study showed an accelerated secretion of angiogenic factors *in vitro* and increased blood perfusion *in vivo* by using a coculture of fibroblasts and human smooth muscle cells [65]. In addition, Sekine et al., showed that a coculture of cardiomyocytes and endothelial cells in a cell sheet enhanced vascularization and that the implanted sheet improved cardiac performance compared with a cardiomyocyte-only sheet [36]. 

Stimulation of angiogenesis has also been shown in single cell-type cell sheets. Miyagawa et al., demonstrated that human HGF gene transfection enhanced the cellular cardiomyoplasty likely by stimulating angiogenesis, restoring the impaired extracellular matrix, and promoting the integration of the dissociated grafted myocytes [66]. Zakharova et al., fabricated sheets from cardiac progenitor cells and showed that these sheets improved cardiac function, suppressed wall thinning, and increased vascular density [35].

To understand the molecular mechanisms of cell sheet angiogenesis Sekiya et al., studied both *in vitro* and *in vivo* models and demonstrated that cardiac cell sheets express VEGF, Cox-2, and Tie-2 and exhibit endothelial cell organisation and microvessel formation [67]. Kitabayashi et al., showed that myoblast sheets express proangiogenic VEGF and placental growth factor and that implantation of these sheets induces angiogenesis *in vivo*. Expression of these proangiogenic genes was further induced by preventing graft apoptosis with antiapoptotic gene therapy [43]. Moreover, Siltanen et al., showed that the proangiogenic effect of myoblast sheets is mediated via the Flt1/Flk-1 pathway [44]. Memon et al., reported expression of SDF-1, HGF, and VEGF in the myocardium after myoblast sheet transplantation [27]. Finally, expression of human HGF in myoblast sheets further enhanced the proangiogenic potential of myoblast sheet therapy [68]. In this study, HGF-expressing sheet therapy increased vascular density in the infarct and border area, as well as in the noninfarcted myocardium.

## 6. Conclusion

Difficulties in reproducibility in cell injection therapy, including low survival and function of the cells, have led to search for more robust methods. Engineering of 3D cell constructs has currently been under extensive investigation. Preassembled cell constructs might provide effective tools for the future cell therapy research. Establishment of programmable materials used in the cell engineering technology has enabled the creation of scaffold-free cell sheets in a rather simple and inexpensive method. The main aspects of cell sheet construction that must be met for successful regenerative therapy are dynamic, electrical, and histological integration. Increased cell-to-cell communication and survival in cell sheets warrant further attention. The reviewed studies demonstrate the existing potential to produce viable, functional myocardial tissue implantable constructs well beyond the current diffusion-limited thickness regime.

## Figures and Tables

**Table 1:** Cell transplantation by intramyocardial injection and vascular infusion in clinical studies.

Cell injection to myocardium				
Cell type	×10^6^ cells	Function	Remarks	
Myoblast	…	↑	Arrhythmogenic potential	[20]
Myoblast	206	No effect	Arrhythmogenic potential	[21]
Myoblast	400–800	No effect	Arrhythmia	[14]
Myoblast	870	…	Arrhythmogenic potential	[22]
Myoblast, BMSC	300, 100	↑		[15]

Vascular infusion				
Cell type	×10^6^ cells	Function	Remarks	

BMSC	*∼*1.5–9	…	Restoration of coronary flow reserve	[12]
BMSC	39	↑		[13]
BMSC	68	No effect		[11]
BMSC	~100–300	↑	Lower mortality	[8]
BMSC	200–400	No effect	Infarct remodelling	[10]
BMSC	56–60 000	↑		[9]

BMSC: bone marrow stem cell.

**Table 2:** Cell types and delivery methods in cardiac functional outcome.

Cell injection to myocardium					
Model	Cell type	×10^6^ cells	Function	Remarks	
Mouse	BMSC	0.0175–0.1	↑	Regeneration	[1]
Rat	BMSC	10	↑	Arrhythmogenicity	[24]
Rat	BMSC	20	↑		[23]
Rat	CM	0.2	↑	Regeneration	[6]
Rat	CM	5	…	Cell washout	[16]
Rat	Myoblast	1	↑	Improved exercise capacity	[28]
Rat	Myoblast	2.3	↑		[29]
Rat	Myoblast	10	↑	Arrhythmogenicity	[23]
Rabbit	FB			↑ diastolic, ↓systolic performance	[30]
Rabbit	Myoblast		↑		[30]
Sheep	Myoblast	…	↑		[31]
Swine	BMSC	100	…	Cell washout	[17]
Swine	BMSC	100	…	High cell homing	[25]

Vascular infusion					
Model	Cell type	×10^6^ cells	Function	Remarks	

Mouse	BMSC	20	…	Regeneration	[3]
Rat	BMSC	10	↑		[24]
Swine	BMSC	100	…	Cell washout	[25]

Cell sheets					
Model	Cell type	Nr of sheets	Function	Remarks	

Hamster	Myoblast	2 layers	↑	Higher life expectancy	[32]
Rat	AdMSC	Monolayer	↑	Paracrine pathways—angiogenesis	[33]
Rat	AdMSC	Monolayer	↑		[34]
Rat	CM, SC	Monolayer	↑	Cell survival, proliferation, differentiation	[35]
Rat	CM, EC	“Monolayer”	↑	Higher vessel density	[36]
Rat	CM	2 monolayers	↑	Integration of graft and myocardium	[37]
Rat	CM	Bilayer		Functional integration	[26]
Rat	CM	3 monolayers	…	Integration of graft and myocardium	[38]
Rat	CM	3 monolayers	…	Thick grafts, multiple surgeries	[39]
Rat	FB, EC	3 monolayers	↑	Less fibrosis	[40]
Rat	Myoblast	1–5 monolayers	↑	Dose-response	[41]
Rat	Myoblast	2 monolayers	↑	Less fibrosis, remodeling	[27]
Rat	Myoblast	2 layers	↑	Less fibrosis, higher vessel density	[42]
Rat	Myoblast	2 × 5 layers	↑	Higher vessel density	[43]
Rat	Myoblast	2 × 5 layers	↑	Paracrine pathways— angiogenesis	[44]
Swine	Myoblast	2–10 layers	↑		[45]

AdMSC: adipose tissue-derived mesenchymal stem cell, BMSC: bone marrow stem cell, CM: cardiomyocyte, EC: endothelial cell, FB: fibroblast, SC: stromal cell.
